# Varicella-Zoster Virus Meningitis and Encephalitis: An Understated Cause of Central Nervous System Infections

**DOI:** 10.7759/cureus.11583

**Published:** 2020-11-20

**Authors:** Jose C Alvarez, Jorge Alvarez, Javier Tinoco, Patricio Mellado, Hector Miranda, Marcela Ferrés, Jonathan Forero, Cristian Álvarez

**Affiliations:** 1 Internal Medicine, University of Antioquia, Medellin, COL; 2 Infectious Disease, Faculty of Medicine, Pontifical Catholic University of Chile, Cali, COL; 3 Infectious Diseases, Pontifical Catholic University of Chile, Cali, COL; 4 Neurology, Pontifical Catholic University of Chile, Cali, COL; 5 Infectious Disease, Pontifical Catholic University of Chile, Cali, COL; 6 General Medicine, University of Antioquia, Medellín, COL; 7 Internal Medicine, University of Sucre, Sincelejo, COL

**Keywords:** varicella zoster virus, meningitis, encephalitis, polymerase chain reaction, central nervous system infection, rash cutaneous lesions

## Abstract

Background

Varicella-zoster virus (VZV) and herpes zoster cause infections of the central nervous system (CNS) manifesting as meningitis or encephalitis. As compared to enterovirus (EV) and herpes simplex virus 1 (HSV-1) and 2 (HSV-2), it is not often tested in CNS infections due to VZV and herpes zoster. There is a certain tendency to think that the findings in the cerebrospinal fluid in infections of the CNS by viruses are comparable among themselves. The exact proportion of patients with VZV primary and reactivation infection who present with lesions prior to or concomitant to its involvement in the CNS is unknown. It is also not known about the risk factors that lead to the reactivation of VZV and CNS involvement.

Objective

To describe the clinical characteristics and laboratory results of patients with a positive VZV polymerase chain reaction (PCR) and neurological signs and symptoms.

Methods

A retrospective and descriptive study was performed at the Hospital Universitario de la Pontificia Universidad Católica de Chile (Hospital Clínico UC CHRISTUS) from September 2012 to July 2014. The following parameters were recorded: neurological signs and symptoms, PCR for VZV in cerebrospinal fluid (CSF), comorbidities, personal medical history, cutaneous lesions, CSF characteristics, CNS imaging, electroencephalography (EEG), treatment, mortality, and neurological sequelae. Adult patients with meningitis, encephalitis, or meningoencephalitis due to VZV diagnosed with PCR were included.

Results

Out of 70 CSF samples analyzed in the previously mentioned period, 21 cases were VZV positive, 16 cases that had clinical information available were included. The mean age with VZV CNS reactivation was 47 years (range 19-80 years). Five patients (31.25%) were immunocompromised: three had human immunodeficiency virus (HIV), one had kidney transplantation, and one had primary immunodeficiency. Clinical presentation was meningitis in 11 patients (68.75%) and encephalitis in five patients (31.25%). Pleocytosis in CSF was observed in all the samples. The five immunocompromised patients had cutaneous lesions. All patients received antiviral treatment. Therapy duration was from 10 up to 21 days. The clinical course was positive in most patients and the mean hospitalization time was 15 days (range 5-60 days). No mortality was observed.

Conclusions

VZV is a worldwide virus and a common cause of CNS infection. The rising incidence is probably due to a better diagnostic method and a frequent clinical suspicion even in the absence of cutaneous lesions, except in immunocompromised cases, as it was observed in the present study. CNS infection presented as a wide spectrum of clinical manifestations with possible neurological sequelae. There was a reduction in neurological morbidity with antiviral therapy. Nonetheless, both the incidence and the morbidity of CNS VZV infection are expected to be diminished by varicella and herpes zoster vaccination. Additionally, there was no increase in mortality in these patients.

## Introduction

Varicella-zoster virus (VZV) causes a disease known as chickenpox when a child is infected, and herpes zoster when reactivation is detected. The main risk factors are elder age and immunosuppression [1]. A central nervous system (CNS) infection presents as a wide spectrum of conditions, such as meningitis, encephalitis, myelopathy, and vasculopathy [2]. Its frequency is higher than previously expected, mostly after polymerase chain reaction (PCR) was introduced as a diagnostic method [3]. VZV-induced meningitis or encephalitis are described both as primary infection and reactivation, frequently associated with cutaneous lesions and poor neurological outcome. Nonetheless, these characteristics have been recently examined; some studies using VVZ PCR have observed up to 55% of cases with CNS infection and no cutaneous lesions associated [4].

However, few epidemiological studies describing clinical characteristics and outcomes are published, and none of them evaluated the Chilean population [5].

Our main objective is to describe the clinical characteristics and laboratory results of patients with a positive VZV PCR and neurological signs and symptoms. In addition, we will analyze whether the presentation of cutaneous lesions previously or in the development of neurological involvement has any relationship with the immune status of the patients. Finally, we performed a literature search for case reports, making reference to the relationship between neurological engagement and VZV diagnosed with PCR.

## Materials and methods

Study design

A retrospective, descriptive study was performed. Adult patients with meningitis, encephalitis, or meningoencephalitis due to VZV diagnosed with PCR were included.

Data collection

Information was retrieved from charts of patients with a VZV CNS infection admitted to the emergency department (ED) in the Hospital Universitario de la Pontificia Universidad Católica de Chile (Hospital Clínico UC CHRISTUS) from September 2012 to July 2014 is located in the center of Santiago de Chile city, with an estimated population of 6,061,185 residents [6]. The decision to perform a PCR was taken by ED doctors. Hence, patients with neurological signs and symptoms and a positive VZV PCR in cerebrospinal fluid (CSF), whose charts were available, were included. Comorbidities, personal medical history, cutaneous lesions, CSF characteristics, CNS imaging, electroencephalography (EEG), treatment, mortality, and neurological sequelae were described.

Case definition

Viral meningitis was defined as the presence of the signs and symptoms of meningitis (fever, headache, meningeal signs), leucocyte count > 5 x 10⁶/L, and negative CSF culture. Encephalitis was defined with similar CSF findings, with additional loss of consciousness, behavioral changes, seizures, focal neurological signs, electroencephalogram (EEG) abnormalities, or CNS imaging compatible with encephalitis (cerebral magnetic resonance imaging (MRI) shows abnormal enhancement or hyperintensity areas in T2W, located in the gray and white matter junction, deep white matter).

Patients were considered immunocompetent when no cellular or humoral immunosuppression due to drugs or diseases was detected.

VZV deoxyribonucleic acid (DNA) extraction, PCR, and melting analysis DNA extraction was performed with the LightMix commercial kit (TIB MOLBIOL, Berlin, Germany). VZV and herpes simplex virus 1-2 (VHS 1-2) real-time PCR was conducted with primer and probes specific for the orf29 and pol genes, respectively. To determine the virus detected, a melting analysis was performed.

## Results

**Figure 1:**
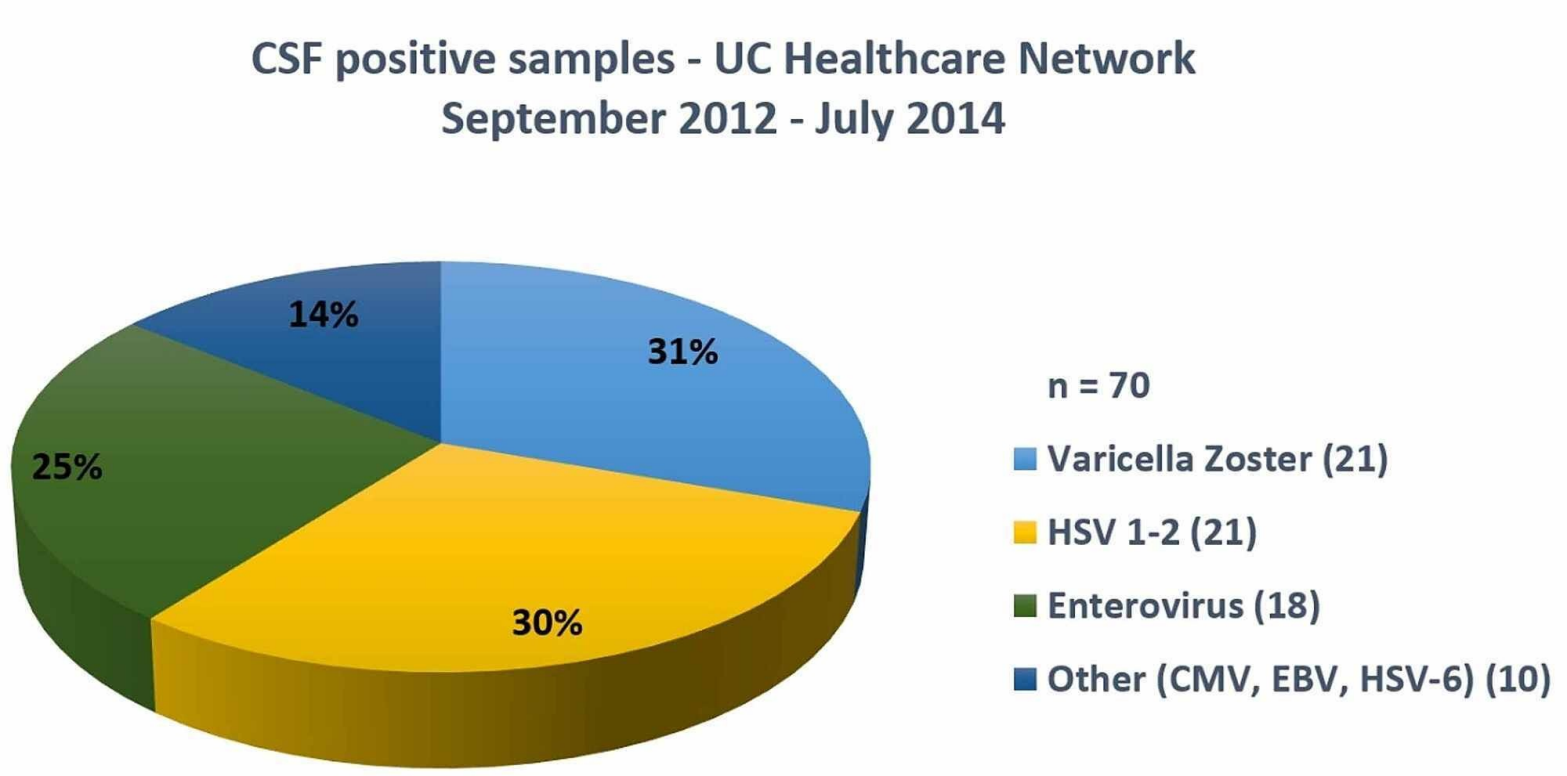
Positive CSF distribution – data from September 2012 to July 2014

Out of 70 CSF samples analyzed in the previously mentioned period, 21 cases were VZV positive. Sixteen cases that had clinical information available were included (Figure 1).

The mean age with VZV CNS reactivation was 47 years (range 19-80 years), with four patients with an age > 60 years. Fourteen patients were male (87.5%). The yearly distribution was as follows: two in 2012 (12.5%), eight in 2013 (50%), and six in 2014 (37.5%) (Table 1). Five patients (31.25%) were immunocompromised: three had HIV, one had kidney transplantation, and one had a primary immunodeficiency.

**Table 1:** Immunocompromised and immunocompetent patient characteristics

Characteristics	Immunocompromised n = 11	Immunocompromised n = 5
Mean age (years)	52.5	39
Male/female	9/2	5/0
Clinical presentation (Meningitis/encephalitis)	6/5	5/0
Cutaneous lesions (%)	0%	100%
Neurological sequelae (%)	18% (mild)	0%

The clinical presentation was meningitis in 11 patients (68.75%) and encephalitis in five patients (31.25%). Pleocytosis in CSF was observed in all the samples. The five immunocompromised patients had cutaneous lesions: two were localized (dorsal or lumbar dermatome) and three were disseminated (more than one dermatome or generalized lesions with no specific distribution) (Table 2). All patients underwent brain MRI. All patients received antiviral treatment: nine with intravenous acyclovir (56.25%) on a 10-15 mg/kg every eight hours schedule and seven with intravenous aciclovir associated with valaciclovir. Therapy duration was from 10 up to 21 days.

**Table 2:** Clinical, laboratory, and imaging findings CSF: cerebrospinal fluid; CNS: central nervous system; HIV: human immunodeficiency virus; CD4: CD4 lymphocytes; M: male; F: female; L: leucocytes; M: monocytes; Lymph: lymphocytes; P: proteins, G: glucose; ADA: adenosine deaminase; DM: diabetes mellitus; IgG: immunoglobulin G; IgA: immunoglobulin A * Not related to hemodialysis ¢ Mild right paresis ŧ Memory loss

Year	Sex	Age (Years)	Clinical presentation	CSF	Cutaneous lesions	Immune state	CNS imaging	Treatment	Outcome
2012	M	36	Meningitis	L28; M 82% P: 77; G 64 ADA 1.4	Yes	HIV CD4: 343	Normal	Aciclovir	Positive
2012	M	56	Meningitis- Cerebral toxoplasmosis	L60; M 100% P 141; G 53 ADA <3.0	Yes	HIV CD4: 54	Frontal and parietal cortical nodules	Aciclovir	Positive
2013	M	56	Meningitis	L:440; M:98% P: 68; G 54 ADA <3.0	Yes	Kidney transplant (2012)	Periventricular and protuberance hyperintensity	Aciclovir Valaciclovir	Positive
2013	M	55	Meningitis	L:30; M 82% P:38; G 38	No	Competent	Normal	Aciclovir Valaciclovir	Positive
2013	M	80	Meningoencephalitis	L:88; M 99% P: 344; G 97 ADA 4,2	No	DM	White matter hypointensity	Aciclovir	Positive Sequelae¢
2013	M	45	Meningitis	L:170; M 100% P:111; G 65	No	Competent	Normal	Aciclovir Valaciclovir	Positive
2013	M	55	Meningitis	L:283; M 1%; Lymph 92% P:140; G 60	No	Competent	Normal	Aciclovir Valaciclovir	Positive
2013	F	51	Meningitis	L:250; M 98% P: 500; G 38	No	Competent	Normal	Aciclovir Valaciclovir	Positive
2013	M	70	Meningoencephalitis	L:310; M 100% P: 320; G122 ADA 4.4	No	DM	Normal	Aciclovir	Positive
2013	M	20	Meningitis	L:440; M 98% P:68; G 54	Yes	Low IgG, IgA	Normal	Aciclovir Valaciclovir	Positive
2014	M	61	Meningoencephalitis	L:165; M 7%; Lymph 92% P:96; G 36 ADA 6.7	No	Chronic kidney disease*	Normal	Aciclovir	Positive
2014	M	19	Meningitis	L:280; M 99% P:126; G 32	No	Competent	Normal	Aciclovir	Positive
2014	F	29	Meningitis	L: 310; M 100% P:65; G 61	No	Competent	Normal	Aciclovir Valaciclovir	Positive
2014	M	27	Meningitis	L:285; M 99% P:157; G 42	Yes	HIV CD4 227	Normal	Aciclovir	Positive
2014	M	52	Meningoencephalitis	L:114; M 99% P:251; G 39 ADA 7	No	Competent	Normal	Aciclovir	Positive Sequelaeŧ
2014	M	61	Meningoencephalitis	L:65:M 7%;Lymph 92% P:92; G 34	No	Chronic kidney disease*	Normal	Aciclovir	Positive

The clinical course was positive in most patients; neurological sequelae were observed in two cases (12.5%): one with memory loss and one with minor right paresis. Both sequelae presented in immunocompetent patients. Mean hospitalization time was 15 days (range 5-60 days). No mortality was observed.

## Discussion

VZV CNS reactivation is associated with a wide range of serious and potentially lethal complications in both immunocompetent and immunocompromised patients, even though it was initially thought of as a mild disease that affected immunocompromised individuals only.

Unlike classical textbooks’ descriptions, the present series is characterized by immunocompetent patients younger than 60 years. Our results are, therefore, comparable to those reported by Persson et al. in Sweden [7], Lozano Becerra et al. in Switzerland [8], and De Broucker et al. in France [9]. Immunocompromised patients were 38%, 18%, and 15%, respectively.

An important aspect of our study is the absence of cutaneous lesions, observed in only 31.5% of our patients as compared to 38% reported by Persson et al., 45% by Lozano Becerra et al., and 45% by De Broucker et al. [7-9]. It is worth noting that our finding was limited to immunocompromised patients only. Therefore, our results of immunocompromise associated with cutaneous lesions nearly duplicate those observed in previously mentioned studies. Traditionally, vesicular lesions are thought of as a sine qua noncharacteristic of viral reactivation. However, plenty of recent studies have confirmed the low sensitivity for these lesions, being absent in up to a third of patients with CNS infection.

CSF pleocytosis was observed in all cases, similar to those previously reported by De Broucker et al. and Persson et al. [7,9]; however, Persson et al. reported 5% patients without pleocytosis, including both immunocompetent and immunocompromised patients. In contrast, Lozano Becerra et al. reported the absence of pleocytosis in nearly 50% of meningoencephalitis patients. This suggests that protecting immune-mediated mechanisms are involved in this inflammatory response.

In our case series, most brain imaging obtained was considered normal, except in a case with periventricular and protuberance hyperintensity. Normal neuroimaging was reported as well by De Broucker et al. and Lozano Becerra et al. [8-9]. Brain MRI in encephalitis usually reveals hyperintense areas in the T2WI sequence, with no enhancement in basal ganglia, caudate nucleus, the internal or external capsule, or the gray and white matter junction.

On the treatment aspect, all of our patients were treated with intravenous acyclovir as was recommended by the Infectious Diseases Society of America (IDSA). This recommendation, however, is based on small case series. No clinical trial with a significant number of patients has established antiviral therapy efficacy to treat VZV meningitis/encephalitis. Even though few studies have shown VZV DNA clearance from CNS when antiviral therapy is used, aciclovir is the standard therapy, as it is used for other clinical features associated with VZV, has a low rate of adverse effects, and the morbidity associated with CNS infection yields higher risk than aciclovir treatment. It is commonly used in a 5-10 mg/kg dose, three times a day for 10-14 days [10].

Neurological outcomes may differ on whether the patient developed meningitis or encephalitis. The meningitis outcome is quite variable. In the few case reports available, the outcome has been from a positive to a poor one. Persson et al. reported neurological sequelae in up to 50% of VZV meningitis patients after a month follow-up [7]. De Broucker et al. reported cognitive impairment and sensitive/motor deficit in up to 45% of patients [9]. Our case series was quite the opposite: neurological sequelae were observed only in two cases (12.5%): mild right paresis and memory loss. Both presented in immunocompetent patients; however, it is worth remarking that these patients presented with clinical features of encephalitis and not meningitis. Lozano Becerra et al. observed a less favorable outcome in patients with clinical features of encephalitis: 18% presented with neuropsychological impairment, aphasia, facial paralysis, and focal seizures [8].

We documented no mortality in our case series. Lozano Becerra et al. reported a VZV encephalitis mortality of 5%-15%, and 33% in encephalitis cases in HIV-positive patients, even after aciclovir treatment [8]. De Broucker et al. observed 15% inpatient mortality and 10% mortality after patients were discharged [9].

Some studies have observed that neurological sequelae are more frequently found in patients with no cutaneous rash [11-12]. The absence of typical cutaneous lesions may lead to delayed treatment and worse outcomes.

During the 1990 decade, VZV CNS complications were considered rare [13]. Diagnosis required both typical cutaneous lesions and specific neurological symptoms. Nevertheless, VZV PCR in cerebrospinal fluid has led to a significant rise in cases, portraying an essential role in CNS infections, particularly in patients with no cutaneous lesions [2].

Data have previously shown that a VZV CNS infection has a relevant frequency. Incidence increases with age: 2.5 cases per 1000 patients in the 21-50 years range as compared to 10.1 cases per 1000 patients in the >80-year range. Furthermore, incidence rates drastically increase in HIV-positive patients: 29.4 cases per 1000 person-year, in comparison to two cases per 1000 person-year in HIV-negative patients [11]. An annual rate of VZV CNS infections of 1.8 cases per 100.000 residents was observed in Sweden [11].

Complications of VZV or herpes zoster CNS infection are poorly studied. Nonetheless, VZV has been reported as the main viral agent of CNS infection in the Swedish population, following tick-borne encephalitis. In patients with VZV CNS infection, VZV DNA has been detected in 0.5%-11.2% CSF samples through PCR [14-15].

VZV CNS infection is a progressive disease that is rarely lethal [16-17]. CNS reactivation portrays a challenge both for inpatient and outpatient physicians. Clinical features are diverse, nonspecific, and indistinguishable from other viral CNS infections. In every patient with encephalitis or suspicion of viral meningitis, VZV detection through PCR is recommended, to establish proper antiviral treatment [18-19].

## Conclusions

VZV is a worldwide virus and a common cause of CNS infection. The rising incidence is probably due to a better diagnostic method and frequent clinical suspicion even in the absence of cutaneous lesions, except in immunocompromised cases, as observed in the present study. Neurological sequelae are not rare and can be serious even after correct treatment and recovery. Even though no clinical trials evaluating antiviral therapy have been performed, a reduction in neurological morbidity has been reported. Nonetheless, both the incidence and morbidity of CNS VZV infection are expected to be diminished by varicella and herpes zoster vaccination.
